# The relationship between evidence-based practices’ facilitators and barriers among nurses and their competencies: self-efficacy as a mediator

**DOI:** 10.1186/s12912-025-02896-2

**Published:** 2025-04-25

**Authors:** Amal Diab Ghanem Atalla, Ayman Mohamed El-Ashry, Samia Mohamed Sobhi Mohamed

**Affiliations:** 1 Department of Nursing Administration, Faculty of Nursing, Alexandria University, Alexandria, Egypt; 2 Department of Psychiatric and Mental Health Nursing, Faculty of Nursing, Alexandria University, Alexandria, Egypt

**Keywords:** Barriers, Competencies, Evidence-based practice, Facilitators, Mediator, Nurses, Self-efficacy

## Abstract

**Background:**

Although evidence-based practices are crucial for enhancing nursing abilities and patient outcomes, many nurses encounter barriers and facilitators that limit their capacity to effectively apply evidence-based practices. Self-efficacy is crucial to how nurses view and overcome these challenges.

**Aim:**

This study aimed to examine the relationship between evidence-based practice facilitators and barriers and nurses’ competencies, with a specific focus on self-efficacy as a mediating factor.

**Design:**

A correlational descriptive design was used.

**Methods and tools:**

A stratified random sampling of 350 nurses provided data for the study using structured questionnaires that assessed evidence-based practice facilitators, barriers, nurses’ self-efficacy, and competencies.

**Results:**

The strongest positive correlation was observed between practice competency and Knowledge (*r* = 0.903, *p* < 0.001), skills (*r* = 0.903, *p* < 0.001), and utilization (*r* = 0.921, *p* < 0.001). On the other hand, EBP barriers show significant negative correlations with attitude (*r* = -0.140, *p* = 0.009), knowledge (*r* = -0.114, *p* = 0.032), and skills (*r* = -0.198, *p* < 0.001).

**Conclusion:**

The findings of this study highlight a significant mediating effect of self-efficacy on the relationship between evidence-based practice facilitators, barriers, and nurses’ competencies.

**Nursing implications:**

The results of this study demonstrate how critical it is to support nurses’ self-efficacy to help them overcome obstacles and improve their capacity to apply evidence-based practices, which will ultimately improve nursing competence and patient care.

**Clinical trial number:**

Not applicable.

## Introduction

A worldwide foundational strategy for delivering standardized care to raise the standard of healthcare based on the most recent scientific findings is known as evidence-based practice (EBP) [1]. EBP is not new in healthcare; in their formative writings, Sackett et al. (1995) define evidence-based medicine (EBM) as follows: “The diligent, clear, and prudent application of the best available information when making decisions regarding the treatment of specific patients is known as evidence-based medicine. In addition to considering patients’ desires and preferences, it combines individual therapeutic knowledge with the best external clinical evidence from systematic research [2].

Also, in 1858, Florence Nightingale brought it to the nursing profession [1, 3]. EBP is similar to a recipe in that patient choices, nursing knowledge, and the most recent research harmonize harmoniously. This combination empowers nurses to make knowledgeable decisions and choose the best courses of action. Keeping the patient’s best interests front and center improves the quality of care and advances nursing. EBP promotes or supports patient-centered nursing care [2, 3]. This emphasis on patient-centered care should reassure the audience about the significant impact of their work on healthcare quality.

The EBM principles are expanded upon by the EBP concept, which applies them to all healthcare professions. EBP promotes collaborative and interdisciplinary approaches to care by emphasizing the integration of patient preferences, clinical competence, and the best available evidence to make well-informed decisions. It acts as a unifying framework that matches the particular objectives and duties of various healthcare positions with the evidence. EBN uses the EBP framework in nursing similarly. With an emphasis on the distinctive features of nursing care, including patient advocacy, holistic care, and the creation of customized care plans, it integrates methodical research, clinical knowledge, and patient values. EBN has been demonstrated to encourage nursing practices based on high-quality evidence, improve clinical outcomes, and increase patient safety.​ By showing how evidence-based practices change to accommodate the demands of many professions and promote interprofessional collaboration, these definitions and their contextual implementations will enhance the study. This inclusion may also help readers better grasp how EBN and EBP uphold the common objective of enhancing patient outcomes while honoring the distinctive contributions of different medical specialists [4].

By ensuring that interventions are founded on the greatest available evidence, the implementation of EBP contributes to improving the quality of care. Additionally, EBP encourages the use of standardized care procedures and minimizes practice variances, which improves patient outcomes and safety. It additionally enhances the professional growth of nurses by encouraging a culture of critical thinking and lifelong learning [3]. The benefits of EBP are hard to summarize because it’s a continuum that’s frequently applied in varying degrees and has barriers that differ depending on the context and discipline. Recognizing relevant barriers and facilitators for EBP could help promote and execute it [5].

Facilitators assist in implementing and maintaining evidence-based practices [6]. A wide variety of experts are involved in facilitating the implementation of EBP. Nurse practitioners (NPs) and clinical nurse specialists (CNSs) are examples of advanced practice nurses who frequently take the lead by mentoring others and advocating for evidence-based practices. EBP specialists, also known as knowledge brokers, are researchers or clinicians with training in implementation science and evidence translation who concentrate on interdisciplinary collaboration to synthesize and apply findings in clinical settings. Academic partners with expertise in project development and evidence evaluation include researchers and faculty from nursing and healthcare schools. To assist the implementation of EBP, organizational leaders including managers and clinical educators, provide training, policy, and tools. Interdisciplinary team members, such as social workers, physical therapists, and pharmacists, also use their specific expertise to customize therapies to fit a range of patient requirements, highlighting a cooperative approach to evidence-based care [7].

Facilitators focus on multiple functions, such as creating a culture that values research, providing training to improve research literacy, developing accessible resources (e.g., summaries or translations of research), and establishing systems that reward the use of evidence-based approaches. Their effectiveness often depends on their ability to communicate research findings, foster collaboration among team members, and adapt evidence to the local context [6, 7].

According to nursing attitudes, skills, and EBP knowledge, nurses encounter several challenges while putting EBP into practice, which reduces their degree of engagement. Lack of scientific understanding and abilities required to convert research into applications and policy, a lack of time, a staffing shortage, a heavy patient workload, and family responsibilities, difficulty understanding statistical analyses, lack of knowledge about EBP, unfavorable opinions about it, and poor academic credentials are among the obstacles and barriers that are commonly mentioned [6, 8–10]. Nurses must be aware of the facilities and obstacles associated with evidence-based practice to address these issues and help them advance their EBP competencies.

EBP competency is the culmination of complicated knowledge, skills, and attitudes, and it is the capacity to formulate clinically pertinent questions to gather, evaluate, apply, and appraise information from a variety of sources while providing care for a specific patient, group, or community. These competencies are primarily concerned with helping nurses build the practice skills necessary to apply EBP. To afford professional nurses, the confidence to carry out EBP activities, researchers stress the necessity of developing nurses’ EBP competencies even at the undergraduate level and focus on developing educational materials and digital technologies [11, 12].

Beliefs in one’s capabilities to organize and execute the courses of action required to produce given attainments” is how Bandura defined self-efficacy [13]. It is a dynamic, context-dependent construct that has a big impact on how people approach tasks, difficulties, and goals. The premise that self-efficacy is a domain-specific belief impacted by four primary sources namely: mastery experiences, vicarious experiences, social persuasion, and physiological/emotional states, rather than a general quality is emphasized by Bandura’s work [13, 14]. These elements are closely related to the particular setting in which self-efficacy is being evaluated, be it education, healthcare, or another domain. Because of this contextual significance, it is crucial to match references to self-efficacy with research that focuses on its use in the pertinent field [13, 14].

The “certainty” that caregivers can perform activities associated with a particular intervention is known as self-efficacy [15]. It has repeatedly been demonstrated that self-efficacy or capability beliefs have a major impact on the encouragement of EBP implementation at the point of care. The association between knowledge and implementation was mediated by self-efficacy, a crucial stage in creating models that support the application of self-efficacy theory to encourage EBP implementation [16].

### Significance of the study

Nationally, an investigation was carried out in Egypt in 2023 of 14 Egyptian Universities and one National Research Centre, including, the faculties of medicine at the universities of Ain Shams, Al-Azhar, Alexandria, Assiut, Benha, Cairo, Helwan, Mansoura, Menoufa, Minia, October 6, Port Said, Suez Canal, and Zagazig, as well as the Ministry of Health [17]. The experience, facilitators, and barriers to using clinical guidelines and EBP were discussed and shared in the previous study. It revealed that personnel with experience and competence in instructing, training, and medical education across a range of contexts and settings are the best facilitators; However, the most significant obstacles were a lack of funding, a lack of national consensus on how to develop, adapt, formally approve, implement, revise, and update EBP as well as constraints on the capacity to carry out research, generate evidence-based recommendations, and efficiently plan and execute EBP [17].

Finding the essential EBP competencies and learning outcomes for European nurses was the goal of an international study in 2021 as the study’s findings suggest that nurse educators, managers, and other EBP stakeholders can create content that integrates EBP knowledge, abilities, and attitudes into educational programs by using the set of EBP competencies and learning objectives as a guide. Healthcare organizations must prioritize EBP competencies in every situation to provide high-quality care and achieve patient satisfaction [18]. Enhancing patient safety, increasing clinical outcomes, and guaranteeing high-quality patient care all depend on having EBP competencies. It emphasizes how important it is for nurses to acquire fundamental EBP skills to successfully participate in healthcare delivery and decision-making [18].

From the researchers’ experience and observation in various healthcare settings adopting EBP there was a need to shed light on how self-efficacy influences the interaction between nurses’ abilities, EBP facilitators, and obstacles. Also, identifying important elements that affect the adoption of EBP.

### Aim of the study

- The study aimed to investigate the relationship between evidence-based practices’ facilitators and barriers among nurses and their competencies: self-efficacy as a mediator.

### The possible research hypotheses are

#### • Hypothesis 1

EBP facilitators are positively associated with nurses’ competencies in EBP.

#### • Hypothesis 2

EBP barriers are negatively associated with nurses’ competencies in EBP.

#### • Hypothesis 3

Self-efficacy mediates the positive relationship between EBP facilitators and nurses’ competencies.

#### • Hypothesis 4

Self-efficacy mediates the negative relationship between EBP barriers and nurses’ competencies.

### Research design

For this study, a descriptive correlational cross-sectional prospective research design was adopted.

### Setting

The investigation was conducted in the Alexandria Main University Hospital, which is connected to Alexandria University. With almost 6760 beds accessible, it provides public health services at no cost to the public. It is the biggest teaching hospital of a university in Alexandria. There were twenty-three critical care units (*N* = 23) in which the study was carried out. These units included the intensive care units for emergency operations, surgical emergencies, burn units, pulmonology units, neurosurgery units (1) and (2), pediatric neurosurgery units, hematemesis units, urology units, anesthesia units, systemic lupus ICU, hepatic transitional ICU, diabetic ICU, and ear, nose, and Trachea (ENT) ICU. Because it is a top healthcare facility with a diversified nursing staff and offers a wide range of clinical services where EBP are crucial, the hospital was selected for this study. Exploring the relationship between EBP facilitators, barriers, competencies, and self-efficacy is made possible by its dedication to professional growth and high-quality service. Furthermore, feasibility and extensive data gathering are ensured by its accessibility and willingness to participate in the study.

### Subjects

415 nurses made up the entire population to determine the sample size. The population had a 95% confidence coefficient, a 50% predicted frequency, and a 5% tolerable error. According to the Epi-Info-7 Program, the fundamental minimum sample size was 256.

**Fig. 1:**
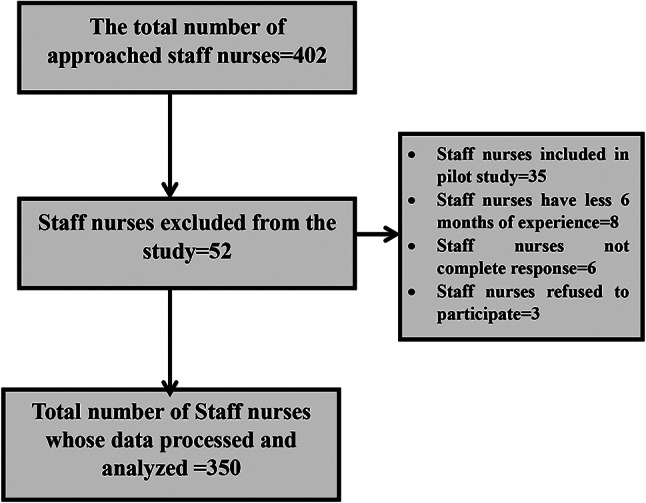
Participants’ recruitment flowchart

Figure 1 flowchart illustrating the recruitment process of staff nurses for the study. 350 nurses who were part of the target group based on the stratified random sampling technique enlisted for this study answered the questionnaire. However, three staff nurses chose not to take part in the study, six nurses were removed for giving partial responses and eight staff nurses had two to four months of work experience in the same unit where they were now employed. All recruited staff nurses must meet the following inclusion requirements: (a) hold a certified staff nurse license, and (b) have worked for a minimum of six months in the same unit as their present job. The study did not include nurses undergoing training or temporarily on leave during the data-collecting period.

### Tools

Four tools will be used in this study as follows:

#### Tool (1): evidence-based practice competency questionnaire, professional version ‘EBP-COQ-Pro

It was developed by Ruzafa-Martínez et al. (2020) to evaluate registered nurses’ skills in connection to EBP. Key dimensions like knowledge, skills, attitudes, and the capacity to use EBP in clinical situations are all assessed by this instrument. It assists in determining nurses’ EBP competencies’ strong points and potential areas for development, directing focused interventions, education, and assistance to strengthen the incorporation of evidence-based practices in healthcare. This tool consisted of a 4-factor model with 35 items [19]. Factor I: attitude (8 items, range 8–40); Factor II: knowledge (11 items, range 11–55); Factor III: skills (6 items, range 6–30); and Factor IV: utilization (10 items, range 10–50) were the four components into which the 35 items were arranged. A Likert scale with a range of 1 to 5 was used to answer the items (a bigger score implies a greater competency). As the EBP-COQ-Pro consists of multiple items grouped into specific domains, such as knowledge, attitudes, skills, and application of EBP. For each domain, the score is calculated by summing the individual item scores within that domain.

The total score is obtained by summing the scores across all domains. This provides an overall measure of the nurse’s EBP competency. Higher scores indicate greater competency in EBP. Domain-specific scores help identify strengths and weaknesses in specific areas (e.g., attitudes toward EBP or practical application skills). The overall score of the level of EBP competency had a range between 35 and 175 points. Internal consistency (Cronbach’s α) for each scale dimension.

was 0.888 for factor I (attitude toward EBP), 0.948 for factor II (EBP knowledge), 0.817 for factor III (EBP skills), and 0.840 for factor IV (EBP utilization).

#### Tool (2): evidence-based practice barrier scale

It was created by Funk et al. and measured obstacles to evidence-based practice (1991). The Barriers Scale is a measurement instrument that was first created for nurses. It comprised 37 measures that looked at how the nurses perceived the three elements (possible barriers): barriers related to nursing (10 items), barriers related to research (14 items), and barriers related to settings (13 items). Scores for the responses ranged from 0 to 4, showing the point to.

according to the degree to which each item hinders evidence-based practice (1, no extent; 2, a little extent; 3, a moderate amount; and 4, a significant extent). A “no opinion” response option received a score of zero [20]. To represent their relative positions on a 0–100% scale, scores between 0 and 4 were transformed into percentiles. The 0th percentile is represented by a score of 0 (“no opinion”), while the 100th percentile is represented by a score of 4 (“a significant extent”). Intermediate scores fall into three equally dispersed categories: the 25th percentile is represented by 1 (“no extent”), the 50th percentile by 2 (“a little extent”), and the 75th percentile by 3 (“a moderate amount”). This mapping offers a consistent method for determining how much each factor impedes evidence-based practice.

#### Tool (3): evidence-based practice facilitators scale

It was developed by Crane (1977). On this scale, nurses are asked to score how much they believe each item supports nurses’ use of research to modify or enhance their practice. There were twelve pieces in all. The scores assigned to the responses ranged from 0 to 4, indicating the degree to which each item is thought to support evidence-based practice (1 being no extent, 2 being somewhat so, 3 being moderately so, and 4 being greatly so). A “no opinion” response option received a score of zero [21]. The degree to which each item is thought to support evidence-based practice is clearly and consistently interpreted by this percentile mapping as follows: 0 (“no opinion”) corresponding to the 0th percentile and 4 (“greatly so”) to the 100th percentile. The intermediate scores were evenly distributed: 1 (“no extent”) corresponds to the 25th percentile, 2 (“somewhat so”) to the 50th percentile, and 3 (“moderately so”) to the 75th percentile.

#### Tool (4) self-efficacy in evidence-based practice (SE-EBP)

Chang & Crowe (2011) developed this tool. It was created to evaluate a person’s assurance and aptitude for providing evidence-based healthcare. The 28 items in this tool were broken down into three categories: identifying the evidence (9 items, α = 0.96), implementing the EBP (14 items, α = 0.96), and identifying the clinical problem (5 items, α = 0.91). The total score is between 0 and 56. Missed care levels vary in intensity: low levels fall between 0 and 18.6, moderate levels between 18.6 and 46.6, and high levels between 46.6 and 56 [22].

The researcher also created a demographic data sheet with questions about age, gender, years of experience as a nurse, working unit, education level, and working unit.

### Ethical considerations

The Nursing Research Ethics Committee of the Faculty of Nursing, Alexandria University in Egypt, accepted the study protocol, confirmed that the investigation followed ethical norms, and provided the reference number IRB00013620/AU/20-8-23. The nurses were informed of the study’s purpose and agreed to participate. Each questionnaire was assigned a code number to protect the respondents’ privacy and identity. The nurses agreed that the data would only be used in the research. Verifying the opt-out option was also essential to ensure the study was conducted ethically. Informed written consent was obtained from nurses before they participated in the study.

### Tools validity

Two natural Arabic speakers translated the tools from English into Arabic. A committee or a third translator examined and clarified the two versions, resulting in a consensus version that correctly mirrored the original meaning. Two translators who had not seen the original English version translated the consensus Arabic version into English. To identify any discrepancies, the back-translated English versions were compared to the original English version. Disagreements were resolved through discussion to ensure that the Arabic translation accurately reflected the source text. A group of seven professors and experts, including multilingual professionals and subject matter specialists (two from mental nursing and five from nursing administration), reviewed the final Arabic translation to ensure its suitability.

### Pilot study

10% of nurses (*n* = 35) agreed with the pilot study’s objective of identifying potential issues and roadblocks during data collection to maintain the goods’ simplicity and usability. There was nothing that needed to be altered. The pilot study data was excluded to maintain a clear distinction between testing and the main study. This decision ensures methodological rigor, avoids bias, and preserves the integrity of statistical analyses. Additionally, as the pilot tested feasibility and adjustments were possible, excluding its data guarantees consistency, ethical transparency, and reliable results in the finalized study.

### Data collection

The questionnaires were distributed directly to the research participants by hand. Each nurse received a copy and was allowed to ask questions during a ten-minute discussion of the study’s aims. After this discussion, the participants were encouraged to complete the questionnaire immediately, which took approximately 15–20 min. The researcher remained available to address any queries during this time. To minimize bias, the participants were assured that their responses would remain confidential and that there were no right or wrong answers. Snacks were provided as a token of appreciation for their time.

The process was carefully planned and executed to ensure efficiency. Because the participants were connected to various functioning units, the researcher coordinated with unit heads to streamline the distribution and collection of completed questionnaires. This approach minimized disruptions to the participants’ workflow and facilitated a high response rate. The data collection period spanned two months, from September to November 2024.

### Data quality management

Throughout the research cycle, the researchers employ various techniques to enhance the general quality of the data. Anomalies, mistakes, redundancies, and inconsistencies must be found and addressed to improve data correctness and integrity. Among other crucial procedures, the process involves data cleansing, validation, profiling, and monitoring.

### Data analysis

Before being incorporated into IBM SPSS version 25, the data was coded. We examined the data’s normality. Inferential statistics, such as ANOVA and the student’s t-test. The demographic and work-related characteristics were measured using quantitative statistics, which include means, frequencies, standard deviations, and percentages. The correlation coefficient examined the connection between evidence-based practices’ facilitators, barriers, competencies, and self-efficacy among nurses. The SEM was employed to determine if self-efficacy may serve as a buffer between evidence-based practices’ facilitators, barriers, and competencies.

## Results

Table 1 shows the demographic data of the 350 nurses. Most (67.7%) were in their 20s, and most were female (80.6%). The marital status was almost evenly split between married (45.7%) and single (51.1%). Regarding qualifications, most were technical nurses (64.9%), and the majority had 1–5 years of nursing experience (57.1%). Most of the nurses had attended training about evidence-based practice (79.1%) and used the Internet in their work (78.0%).

**Table 1:** Distribution of the studied nurses according to demographic data (*n* = 350)

Demographic characteristics	No.	%
**Age (years)**		
20–	237	67.7
30–	63	18.0
40–	39	11.1
≥ 50	11	3.1
Mean ± SD	30.8 ± 6.5	
**Sex**		
Male	68	19.4
Female	282	80.6
**Marital status**		
Married	160	45.7
Single	179	51.1
Widowed	6	1.7
Divorced	5	1.4
**Qualification**		
Professional nurse	66	18.9
Technical nurse	227	64.9
Practical nurse	57	16.3
**Job position**		
Word nurse	22	6.3
ICU nurse	79	22.6
CCU nurse	41	11.7
Pediatric nurse	66	18.9
OR nurse	35	10.0
Emergency nurse	95	27.1
Others	12	3.4
**Experience year of nursing**		
< 1	0	0.0
1–5	200	57.1
5–10	95	27.1
10–15	43	12.3
15 - <20	7	2.0
≥ 20	5	1.4
Mean ± SD	4.3 ± 2.8	
**Experience hospital**		
< 1	45	12.9
1–5	217	62.0
5–10	53	15.1
10–15	24	6.9
15 - <20	6	1.7
≥ 20	5	1.4
Mean ± SD	3.1 ± 2.2	
**Are you attend any training about evidence-based practice**		
Yes	277	79.1
No	73	20.9
**Do you use of the Internet and other digital tools**		
Yes	273	78.0
No	77	22.0

Table 2 shows the distribution of nurses according to their levels and means percent score of attitude, knowledge, skills, and various barriers related to EBP. Most nurses exhibited a high attitude towards EBP, with 58.6% scoring in the high category and a mean percent score of 68.72%. Knowledge and skills were similarly distributed, with nearly equal proportions of nurses in the moderate and high categories, though there was room for improvement as reflected by their mean percent scores of 63.09% and 62.91%, respectively. Regarding utilization, most nurses (57.4%) demonstrated high levels of applying their Knowledge and skills in practice, with a mean percent score of 64.92%. Regarding EBP competency, over half of the nurses (52.3%) scored in the high category, indicating a generally competent workforce with a mean percent score of 65.39%. The overall barriers scale indicated that nearly two thirds (64.6%) of nurses reported moderate levels of EBP barriers; with the high percent of them (73.4%) reported high levels of research barriers. Despite these challenges, the Facilitators Scale shows that 86.9% of nurses reported high level. Self-efficacy was also strong, with nurses feeling confident in their ability to implement EBP, as indicated by all nurses reported moderate to high levels (49.4% and 50.6%, respectively).

**Table 2:** Distribution of the studied nurses according to their levels and mean percent score (*n* = 350)

	Low (< 33.3%)		Moderate (33.3– <66.6%)		High (≥ 66.6%)		Total score	Mean percent score	Mean score
	No.	%	No.	%	No.	%	Mean ± SD	Mean ± SD	Mean ± SD
Attitude	5	1.4	140	40.0	205	58.6	29.99 ± 4.71	68.72 ± 14.70	3.75 ± 0.59
Knowledge	26	7.4	161	46.0	163	46.6	37.62 ± 3.86	63.09 ± 19.31	3.52 ± 0.77
Skills	30	8.6	158	45.1	162	46.3	22.58 ± 4.09	62.91 ± 20.45	3.52 ± 0.82
Utilization	15	4.3	134	38.3	201	57.4	41.58 ± 4.42	64.92 ± 18.41	3.60 ± 0.74
**Tool (I): Evidence-Based Practice Competency Questionnaire**,** Professional version ‘EBP-COQ-Pro**	7	2.0	160	45.7	183	52.3	141.77 ± 15.39	65.39 ± 16.03	3.62 ± 0.64
Nurses. barriers	6	1.7	156	44.6	188	53.7	29.78 ± 4.81	68.09 ± 15.03	2.26 ± 0.61
Research barriers	7	2.0	86	24.6	257	73.4	47.41 ± 4.95	73.15 ± 15.47	2.39 ± 0.67
Settings barriers	15	4.3	138	39.4	197	56.3	41.38 ± 5.34	66.91 ± 16.59	2.72 ± 0.61
**Tool (II): Barrier Scale**	7	2.0	226	64.6	117	33.4	118.57 ± 13.51	61.48 ± 13.29	2.46 ± 0.53
**Tool (3): Facilitators Scale**	0	0.0	46	13.1	304	86.9	37.17 ± 5.66	77.45 ± 11.80	3.10 ± 0.47
**Tool (4) Self Efficacy**	0	0.0	177	50.6	173	49.4	206.63 ± 51.80	73.79 ± 18.50	7.38 ± 1.85

SD: Standard deviation

In Table 3, Practice competency showed a significant positive correlation with EBP facilitators (r = 0.570, p < 0.001) and self-efficacy (r = 0.224, p < 0.001), while it was negatively correlated with EBP overall barriers (r = -0.177, p = 0.001). Among specific barriers, nurses’ barriers (r = -0.149, p = 0.005), research barriers (r = -0.158, p = 0.003), and settings barriers (r = -0.166, p = 0.002) negatively correlated with practice competency. Self-efficacy positively correlated with EBP facilitators (r = 0.354, p < 0.001) but negatively correlated with EBP overall barriers (r = -0.193, p < 0.001), research barriers (r = -0.250, p < 0.001), and settings barriers (r = -0.127, p = 0.017). Strong positive correlations were observed between attitude, knowledge, and skills, and these variables also positively correlated with utilization (attitude and expertise, r = 0.761, p < 0.001; knowledge and skills, r = 0.758, p < 0.001; skills and utilization, r = 0.842, p < 0.001). Barriers, including nurses’, research, and settings barriers, were positively correlated but negatively associated with attitude, knowledge, and skills. The EBP facilitators scale was positively correlated with attitude (*r* = 0.497, *p* < 0.001), knowledge (*r* = 0.507, *p* < 0.001), and skills (*r* = 0.532, *p* < 0.001) and negatively correlated with EBP barriers (*r* = -0.169, *p* = 0.002).

**Table 3:** Correlation between the studied variables (*n* = 350)

		Attitude	Knowledge	Skills	Utilization	Practice Competency	Nurses. barriers	Research barriers	Settings barriers	Barrier Scale	Facilitators Scale	Self-Efficacy
Attitude	r											
	p											
Knowledge	r	0.761*										
	p	< 0.001*										
Skills	r	0.670*	0.758*									
	p	< 0.001*	< 0.001*									
Utilization	r	0.718*	0.759*	0.842*								
	p	< 0.001*	< 0.001*	< 0.001*								
Practice Competency Questionnaire	r	0.881*	0.903*	0.903*	0.921*							
	p	< 0.001*	< 0.001*	< 0.001*	< 0.001*							
Nurses. barriers	r	-0.128*	-0.089	-0.142*	-0.173*	-0.149*						
	p	0.016*	0.098	0.008*	0.001*	0.005*						
Research barriers	r	-0.112*	-0.109*	-0.205*	-0.147*	-0.158*	0.663*					
	p	0.036*	0.041*	< 0.001*	0.006*	0.003*	< 0.001*					
Settings barriers	r	-0.135*	-0.108*	-0.182*	-0.170*	-0.166*	0.776*	0.653*				
	p	0.012*	0.043*	0.001*	0.001*	0.002*	< 0.001*	< 0.001*				
Barrier Scale	r	-0.140*	-0.114*	-0.198*	-0.183*	-0.177*	0.907*	0.862*	0.912*			
	p	0.009*	0.032*	< 0.001*	0.001*	0.001*	< 0.001*	< 0.001*	< 0.001*			
Facilitators Scale	r	0.497*	0.507*	0.532*	0.521*	0.570*	-0.128*	-0.148*	-0.174*	-0.169*		
	p	< 0.001*	< 0.001*	< 0.001*	< 0.001*	< 0.001*	0.017*	0.005*	0.001*	0.002*		
Self-Efficacy	r	0.196*	0.231*	0.212*	0.173*	0.224*	-0.142*	-0.250*	-0.127*	-0.193*	0.354*	
	p	< 0.001*	< 0.001*	< 0.001*	0.001*	< 0.001*	0.008*	< 0.001*	0.017*	< 0.001*	< 0.001*	

r: Pearson coefficient *: Statistically significant at *p* ≤ 0.05

Table 4 revealed the multivariate linear regression analysis showed that practice competency is highly influenced by both obstacles and self-efficacy. Practice competency and barriers had a negative relationship (B = -0.158, *p* = 0.009), suggesting that lower competency results from higher barriers. Conversely, there was a positive effect of self-efficacy (B = 0.059, *p* < 0.001), indicating that practice competency is improved by higher levels of self-efficacy. Although both associations were statistically significant, the model only accounted for 6.9% of the variation in practice competency (R2 = 0.069), suggesting that competency may also be influenced by other factors.

**Table 4:** Multivariate linear regression analysis for factors affecting practice competency (*n* = 350)

Variable	B	Beta	t	*p*	95% CI	
					LL	UL
Barrier Scale	-0.158	-0.139	-2.625*	0.009*	-0.276	-0.040
Self-Efficacy scale	0.059	0.198	3.742*	< 0.001*	0.028	0.090
R^2^ = 0.069, Adjusted R^2^ = 0.063, F = 12.812^*^,*p* < 0.001^*^						

F, p: f and p values for the model

R^2^: Coefficient of determination

B: Unstandardized Coefficients

Beta: Standardized Coefficients

t: t-test of significance

LL: Lower limit UL: Upper Limit

*: Statistically significant at *p* ≤ 0.05

**Fig. 2:**
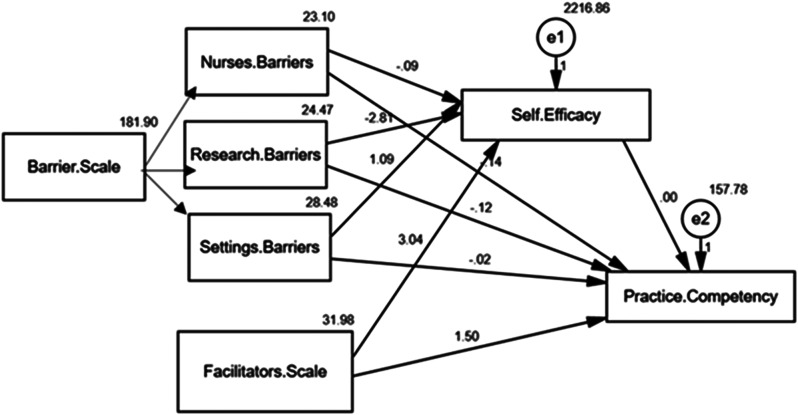
A path analysis of the direct and indirect effect of the EBP Barriers and Facilitators on nurses’ Competencies as mediated by Self-Efficacy

Table 5; Fig. 2 examine the direct and indirect effects of barriers and facilitators on nurses’ practice competency, with self-efficacy as a mediating factor. The results show that self-efficacy has a strong positive direct effect on practice competency, with an estimate of 3.040 (C.R. = 6.706, *p* < 0.001), indicating that nurses who feel more confident in their abilities tend to demonstrate higher levels of practice competency. The Facilitator Scale also has a significant positive direct effect, estimated at 1.504 (C.R. = 11.705, *p* < 0.001), suggesting that supportive factors within the environment contribute positively to practice competency. Conversely, barriers related to nurses, settings, and research all show negative direct effects on practice competency, with estimates of -0.145 (C.R. = -5.616, *p* = 0.001), -0.022 (C.R. = -13.210, *p* < 0.001), and − 2.808 (C.R. = -3.939, *p* = 0.002) respectively, with research barriers having the most substantial negative impact.

**Table 5:** The direct and indirect effect of barrier and facilitators scale on practice competency questionnaire mediating by Self-Efficacy

			Direct effect	Indirect effect	Estimate	S.E.	C.*R*.	*P*
Practice Competency	<---	Self-Efficacy	3.040	-	1.857	0.453	6.706*	< 0.001*
Practice Competency	<---	Facilitators Scale	1.504	0.010	1.749	0.128	11.705*	< 0.001*
Practice Competency	<---	Nurses Barriers	-0.145	0.040	-2.156	0.235	-5.616*	0.001*
Practice Competency	<---	Settings Barriers	-0.022	0.010	-2.421	0.211	-13.210*	< 0.001*
Practice Competency	<---	Research Barriers	-2.808	-0.110	-3.978	0.713	-3.939*	0.002*

Model fit parameters CFI; IFI; RMSEA (1.000; 1.000; 0.116)

Model χ^2^/df. 72.620/3 *p* ≤ 0.001

CFI: Comparative Fit Index, IFI: Incremental Fit Index, RMSEA: Root Mean Square Error of Approximation

Additionally, Table 5; Fig. 2 show that facilitators and barriers indirectly affect practice competency through their influence on self-efficacy. For instance, the Facilitators Scale has a small positive indirect effect (0.010), while Nurses Barriers have a negative indirect effect (-0.040). The settings and research barriers also show indirect effects of 0.010 and − 0.110, respectively. Based on that, self-efficacy partially mediates the relationship between Evidence-Based Practices Facilitators and Barriers among Nurses and their competencies. The model fit parameters (CFI = 1.000, IFI = 1.000, RMSEA = 0.116, and χ²/df = 72.620/3, *p* ≤ 0.001) indicate that the model fits the data well, although the RMSEA value suggests a slightly higher error of approximation.

## Discussion

The implementation of evidence-based nursing practice has emerged as a key issue for clinical nursing practice and the healthcare system. Globally, there is an increasing need to provide high-quality nursing care in the current healthcare system [23]. Although evidence-based practices (EBPs) are becoming more widely acknowledged for their role in enhancing healthcare outcomes, little research has been done on how facilitators and barriers affect nurses’ ability to apply EBPs. Self-efficacy’s involvement in improving practice competency has been investigated in individual research, but its mediating role between EBP facilitators, barriers, and competency is getting less attention. Moreover, most current research concentrates on either facilitators or barriers alone, without a thorough understanding of how these elements combine to influence competency [24]. Therefore, this study aims to identify the relationship between evidence-based practices’ facilitators and barriers among nurses and their competencies with the mediating role of self-efficacy.

This study revealed the strongest positive correlation between EBP competencies and key variables such as attitude, Knowledge, skills, and utilization. This indicates that higher attitude, Knowledge, skills, and utilization levels were closely associated with greater EBP competencies. EBP can be implemented and used more successfully by nurses who possess advanced abilities, positive attitudes, and a greater degree of knowledge. This demonstrates the necessity of thorough instruction and assistance to improve these crucial areas and, eventually, EBP expertise.

Additionally, most nurses exhibited a high attitude towards EBP, which indicates that attitude was closely linked with better Knowledge, skills, and application in practice. This is because most nurses expressed gratitude for the availability of scientific data to support their healthcare. Nurses believed EBP facilitates clinical decision-making and raises the nursing profession’s autonomy. This result was compatible with many studies that showed a significant relationship between nurses’ Knowledge, perception, and attitude toward evidence-based practice. Additionally, a favorable attitude toward EBP and attained intermediate ratings in terms of the application of EBP-related Knowledge and abilities [25–27].

This study showed facilitator factors have a significant positive direct effect on EBP competency. The strong positive direct impact of facilitator factors on EBP competency emphasizes how important systemic and organizational support are to improving evidence-based practice. Effective development and application of EBP competencies by nurses is facilitated by elements including training opportunities, supportive leadership, and resource accessibility. This emphasizes how crucial it is to create supportive conditions to encourage the use of EBP in clinical practice. This reflects facilitator factors, which enhance knowledge, skills, utilization, and overall competency in EBP. For example, increasing research awareness transduces into a common mother tongue, rewarding research users for their efforts, and lengthening the time frame for research results. This conclusion is further supported by Hosseini-Moghaddam et al. (2023), who identify important facilitator characteristics that empower nurses and foster an atmosphere that supports the development of EBP competency. These factors include managerial support, evidence-based nursing committees, and time set aside for research. Their results, which show alignment rather than contradiction, support our thesis by emphasizing the useful facilitators that result in increased competency [28].

In addition, this study revealed that the barrier scale was negatively correlated with attitude, knowledge, and skills. This indicates that higher perceived barriers were associated with lower attitudes, Knowledge, and skills. Furthermore, 73.4% of nurses 73.4% had a research barrier followed by setting or organizational barriers, and both had indirect effects on practice competency through their influence on self-efficacy, as shown in Fig. 2. The researchers rationalized this result by the assumption that the influence of barriers on nurses’ confidence and ability to use evidence-based practices may be the cause of the negative association shown between perceived barriers and EBP attitudes, knowledge, and abilities. Research obstacles can make it difficult to learn and use EBP effectively, such as restricted access to resources or a lack of training. Time restrictions or a lack of assistance are examples of organizational hurdles that can produce a tense atmosphere that lowers motivation and breeds feelings of inadequacy. The ability of nurses to apply EBP in their practice may be indirectly impacted by these difficulties since they can weaken self-efficacy, which is essential for developing competency.

This study is compatible with Pitsillidou et al. (2023), who revealed the absence of administrative support, the challenge of obtaining data, the inadequate Knowledge and attitude of nurses, and the impossibility of safeguarding decisions made were all obstacles to the adoption of Evidence-Based Practice in nursing [29]. Moreover, Shazly et al. (2018) revealed that the EBP Barriers Scale and the EBP Beliefs had a negative relationship [30].

Self-efficacy is a mediating factor and it has a positive direct effect correlation with key variables, particularly Knowledge and practice competency, while it negatively correlates with barriers, such as research barriers. This indicates that higher self-efficacy is associated with better Knowledge, competency, and fewer perceived barriers. That indicates that nurses who feel more confident in their abilities tend to demonstrate higher levels of practice competency. For recently graduated nurses, increasing self-efficacy is essential since it has a big impact on their capacity to function well in the workplace. By including instruction that prioritizes the growth of abilities, knowledge, and confidence, nursing schools can significantly contribute to the development of self-efficacy. Nursing programs can help new nurses understand how their beliefs in their abilities affect their performance and patient outcomes by teaching students about the concept of self-efficacy—its definition, sources (including mastery experiences, vicarious learning, social persuasion, and emotional states), and its impact on professional practice. Self-efficacy and competency can be further improved through instructional methodologies that incorporate mentorship, simulation, and opportunities for reflective practice. In addition to fostering nursing proficiency, this strategy encourages professional development, career happiness, and personal growth—all of which are critical for keeping nurses in the workforce and advancing the field [14].

This result was compatible with Zia et al. (2022), who revealed a positive correlation between EBP and self-efficacy [31]. Additionally, Azmoude et al. (2017) revealed implementation, knowledge, and mean self-efficacy ratings were found to be significantly correlated [32]. Moreover, Dagne et al. (2021) showed participants’ knowledge (AOR = 3.06, CI 1.6–5.77) and self-efficacy of applying evidence-based practice skills (AOR = 12.5, CI 5.7–27.5) were statistically significantly related factors of evidence-based practice implementation [33].

On the other hand, these results are congruent with Song (2024), who discovered that by favorably influencing their EBP views, self-efficacy had an indirect impact on EBP execution intentions [34]. Additionally, according to Clark et al. (2024), the use of EBP was not significantly impacted by learning experiences, self-efficacy, or EBP knowledge [35].

### Strengths and limitations

The research has advantages and is theoretically sound. This gives the research a well-defined conceptual base. This multidimensional approach provides a more thorough knowledge of the complex elements of nursing involvement; the study looks at both barriers and facilitators to EBP implementation. Also, examining self-efficacy as a mediating variable provides information on the psychological mechanisms that underlie the correlation between nurses and EBP variables. The results can guide the creation of focused interventions that raise nurse nurses’ efficacy and, in turn, enhance the application of EBP and patient outcomes.

However, this study has some limitations and shortcomings that should be considered. The results may not be as applicable to different nurse populations or situations if the sample is restricted to a particular area or healthcare facility. Also, it is possible that some confounding factors that could affect how the variables of interest relate to one another were overlooked in the study. Another limitation is the absence of longitudinal data and deeper insights into the dynamics of EBP implementation, which may be gained by examining how the associations change over time, which is impossible with the cross-sectional methodology. Organizational, cultural, and environmental factors may affect nursing involvement and self-efficacy, but this study may not fully capture them. The validity of the results could be impacted by biases like social desirability or recall bias if self-reported data from nurses is used.

## Conclusion

This study provides insightful information about the intricate connections among nurses’ EBP competencies, barriers, facilitators, and self-efficacy. According to the results, self-efficacy plays a critical mediating role in bridging the gap between nurses’ perceived facilitators and barriers to implementing EBP and their competencies. Higher self-efficacy is generally reported by nurses who encounter more facilitators and fewer barriers, and this is positively correlated with increased EBP competency. These findings highlight how crucial it is to promote self-efficacy to strengthen EBP implementation and advance the nursing profession. The nursing profession stands out for its human-centered approach and ethical dedication to patient care. Healthcare organizations should foster a culture of support, continuous education, and communication to empower nurses [36–38]. Encouraging nurses’ active involvement in clinical decision-making with EBP improves patient outcomes and nurses’ self-efficacy.

### Recommendations of the study

To improve EBP competency, this study emphasizes the significance of empowering nurses and creating a supportive workplace. In addition to providing ongoing direction through coaching and mentoring programs, hospital administrators should put in place focused training programs to increase nurses’ proficiency, expertise, and self-assurance in EBP. For nurses to actively participate in EBP, it is essential to create a culture that values this process. Implementing EBP can be made easier by addressing obstacles including a lack of resources, time constraints, or organizational support. Fostering sustainable EBP practices requires policies that minimize administrative hassles, set out protected time for EBP activities, and guarantee access to nurses, modern resources, and technology. To further EBP initiatives, cooperation, and frequent assessment are also essential.

Fostering collaborations between nurses, doctors, and other medical specialists can improve information exchange and the collaborative creation of evidence-based solutions. Encouraging interprofessional education and collaboration facilitates the successful resolution of challenging healthcare issues. To integrate different viewpoints and areas of expertise and provide more thorough evidence-based solutions, it is beneficial to support interprofessional education and collaboration in EBP. This method improves patient outcomes, guarantees successful implementation, and strengthens decision-making.

To determine their impact and direct changes, EBP methods must also be continuously evaluated utilizing both qualitative and quantitative data; EBP techniques can be assessed statistically using metrics like adherence rates, patient outcomes, and statistical comparisons to gauge efficacy and impact, and qualitatively using focus groups, interviews, and thematic analysis to comprehend experiences and obstacles. A thorough evaluation is ensured by combining these methods. To keep projects relevant to nurses’ requirements and flexible enough to meet the changing demands of healthcare, feedback mechanisms should be established to gather their perspectives on barriers, facilitators, and results.

### Future studies

Future studies should examine the dynamics of these interactions over time and the contextual elements that affect how EBP barriers, facilitators, self-efficacy, and nurses interact. A mixed-methods approach that includes qualitative and quantitative data may help us better understand this important aspect of nursing practice. In addition, further studies could be conducted to investigate leadership styles’ effect on nurses’ evidence-based practice competencies; this study could look into how nurses’ EBP competencies, attitudes, and utilization are affected by various leadership philosophies, such as transformational or transactional leadership. Also, the role of digital tools and technology in enhancing evidence-based practice competency could be assessed in how well online training courses, mobile applications, and digital platforms improve nurses’ understanding, proficiency, and application of EBP in a range of healthcare contexts.

## Data Availability

The datasets generated during and analyzed during the current study are available from the corresponding author upon reasonable request.
